# Human Presence Increases Parasitic Load in Endangered Lion-Tailed Macaques (*Macaca silenus*) in Its Fragmented Rainforest Habitats in Southern India

**DOI:** 10.1371/journal.pone.0063685

**Published:** 2013-05-22

**Authors:** Shaik Hussain, Muthuvarmadam Subramanian Ram, Ajith Kumar, Sisinthy Shivaji, Govindhaswamy Umapathy

**Affiliations:** 1 Laboratory for the Conservation of Endangered Species, CSIR-Centre for Cellular and Molecular Biology, Hyderabad, India; 2 Centre for Wildlife Studies, Wildlife Conservation Society-India, Bangalore, India; Texas Tech University, United States of America

## Abstract

**Background:**

Understanding changes in the host-parasite relationship due to habitat fragmentation is necessary for better management and conservation of endangered species in fragmented landscapes. Pathogens and parasites can pose severe threat to species in restricted environments such as forest fragments where there is increased contact of wildlife with human and livestock populations. Environmental stress and reduced nutritional level in forest fragments can influence parasite infection and intensity on the native species. In this study, we examine the impact of habitat fragmentation on the prevalence of gastrointestinal parasites in lion-tailed macaques in a fragmented rainforest in Western Ghats.

**Methods:**

The prevalence of different gastrointestinal parasites was estimated from 91 fecal samples collected from 9 lion-tailed macaque groups in nine forest fragments. The parasites were identified up to genus level on the basis of the morphology and coloration of the egg, larva and cyst. The covariates included forest fragment area, group size and the presence/absence of human settlements and livestock in proximity. We used a linear regression model to identify the covariates that significantly influenced the prevalence of different parasite taxa.

**Results:**

Nine gastrointestinal parasite taxa were detected in lion-tailed macaque groups. The groups near human settlements had greater prevalence and number of taxa, and these variables also had significant positive correlations with group size. We found that these parameters were also greater in groups near human settlements after controlling for group size. Livestock were present in all five fragments that had human settlements in proximity.

**Conclusion:**

The present study suggests that high prevalence and species richness of gastrointestinal parasites in lion-tailed macaque groups are directly related to habitat fragmentation, high anthropogenic activities and high host density. The parasite load partially explains the reason for the decline in immature survival and birth rate in small and isolated rainforest fragments in Anamalai Hills.

## Introduction

Changes in the gastrointestinal parasitic profile of animals due to habitat fragmentation can adversely impact the survival of remnant populations of endangered species and can have implications for human health [1], [2]. Host density and edge effect are two major factors that influence the parasitic profiles in mammalian hosts in fragmented habitats. Host density is a major determinant of the prevalence and species richness of directly transmitted parasites [3], [4]. High host densities lead to higher transmission rates of directly transmitted parasites, some of which might in fact have low prevalence and thus need high transmission rates for their persistence [5], [3]. High host densities also increase the repeated use of the same area and thus can increase their contact with substrates where infective stages of parasites are deposited. Since host densities are often higher in forest fragments in the short term, a higher prevalence and species richness among parasites are expected. In group living animals, group size is equivalent to host density, and parasitic load increases with group size [6], [7]. The loss of canopy contiguity in forest fragments can further exacerbate this for arboreal mammals, as they are forced to spend more time on the ground. Increased host density in fragments can cause social as well as nutritional stress among the hosts, making them even more susceptible to parasitic infection [8]. The lack of potential sleeping sites might also influence parasitic load, since primates might be avoiding infestation by rotating sleeping sites [9].

A greater perimeter to area ratio in forest fragments had influenced more cross-species infection of parasites, as new hosts with different sets of parasites infiltrate the fragments through the edge. Human beings and their livestock are such frequently encountered new hosts. For example, colobus monkeys near the edge of forest have more gastrointestinal parasites compared to those in the interior forests [10]. Host density and edge effect can act synergistically to increase parasitic infection. For example, increased host densities and habitat degradation can increase the interspecies infection of parasites [11].

Changes in parasitic infestation due to habitat fragmentation have been examined in terms of species richness, overall prevalence and intensity. However, there is evidence that different parasitic taxa respond differently to habitat fragmentation. For example, it has been suggested that directly transmitted parasites might benefit from overcrowding than indirectly transmitted ones [12]. Therefore, it is important to examine the infestation patterns of parasitic taxa separately [13].

The lion-tailed macaque, one of the most endangered primate species in the world, is endemic to the rainforest of the Western Ghats mountain range along the western coast of south India. It is a good model species to examine changes in parasitic profiles due to habitat fragmentation. Its habitat is among the most fragmented and densely populated biodiversity hotspots [14]. The population of lion-tailed macaque itself is heavily fragmented, with nearly 40% of the population occurring as small isolated populations [15]. It occurs in higher densities as well as group size in forest fragments than in contiguous forests [16]. There is a negative correlation between fragment area and habitat degradation as indicated by tree densities, basal area and canopy height [16] and the smaller fragments are more likely to have human settlements nearby. Lion-tailed macaques in forest fragments also spend more time on the ground compared to those in contiguous forests [17] and feed on far fewer plant species [18]. Thus by all criteria, we should expect an increased parasitic infection in the lion-tailed macaques, caused by increased host densities and higher cross-species infestation. In this paper we test this hypothesis and also examine differences among parasitic taxa.

## Results

**Figure 1:**
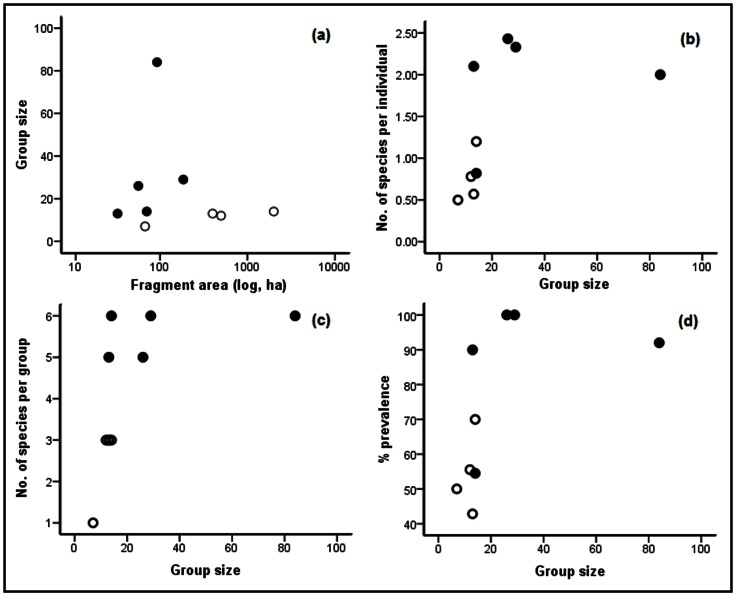
The relationship between group size in the lion-tailed macaque and attributes of gastrointestinal parasites; (a) group size and fragment area; (b) the number of parasitic taxa per individual and group size; (c) the number of parasitic taxa per group and group size; and (d) group size and the overall parasite prevalence in groups in nine forest fragments (•) with or (O) without human settlements.

There was no correlation between group size and fragment area because groups in the smaller fragments were highly variable (*r_s_* = 0.008, P = 0.98; Figure 1a). Although fragments with human settlements were generally smaller, this difference was not significant (Mann-Whitney U = 3, P = 0.11). Human settlement in the periphery or inside the fragment was taken as a proxy for livestock grazing since all five fragments with human settlements also reported livestock grazing inside the fragment (Table 1). We could not, however, obtain reliable estimates of the number of livestock either through direct observation or through interviews. The number of livestock, mostly cattle, did not seem to vary substantially among human settlements and was <50 heads. Therefore, the linear regression model included the prevalence of different taxa as response variables, and group size and presence or absence of human settlement as covariates.

**Table 1:** Habitat attributes of the nine sampled forest fragments and the prevalence and taxon richness of gastrointestinal parasites in lion-tailed macaque (+, present; −, absent).

Fragment name	Fragment area (ha)	Human settlement	Live- stock	Group size	No. of samples	Prevalence (%)	Taxon richness
Varattuparai	24	+	+	13	10	90.0	5
Korangumudi	35	+	+	26	7	100.0	5
Pannimedu	50	−	−	7	6	50.0	1
Puthuthottam	65	+	+	84	25	92.0	6
Sivamalai	70	+	+	14	11	54.5	6
Andiparai	185	+	+	29	6	100.0	6
Urulikkal	500	−	−	12	9	55.7	3
Shekkalmudi	400	−	−	13	7	42.8	2
Akkamalai	2000	−	−	14	10	70.0	3

### Parasite Prevalence

We collected 91 fecal samples from nine lion-tailed macaque groups in 9 forest fragments (Table 1). Overall 75.8% of 91 samples had at least one parasite, the prevalence in a group varying from 42% to 100%. We detected 9 parasite taxa, consisting of 5 nematodes (*Ancylostoma, Bunostomum, Haemonchus, Strongyloides* and *Trichuris*), 2 cestodes (*Diphyllobothrium* and *Moniezia*), one each of trematode (*Cotylophoron*) and protozoan (*Balantidium*) (Table 2). *Strongyloides* was the most prevalent parasite found in 8 forest fragments followed by *Trichuris* and *Ancylostoma* found in 7 forest fragments. Both the cestodes were found in only one forest fragment. Out of 9 taxa identified, 6 occurred near human settlements and 3 occurred in both (Table 2). Of the 3 occurring in both, 2 had higher prevalence near settlements. All the rare ones were found only in fragments near settlements.

**Table 2:** The prevalence of different taxa of gastrointestinal parasites in lion-tailed macaques in the nine rainforest fragments.

Fragment name	Prevalence (%)								
	*Ancylostoma*	*Bunostomum*	*Haemonchus*	*Strongyloides*	*Trichuris*	*Cotylophoron*	*Moniezia*	*Diphyllobothrium*	*Balantidium*
Varattuparai	50.0	40.0	0	30.0	10.0	80.0	0	0	0
Korangumudi	14.3	85.7	0	71.4	57.1	14.3	0	0	0
Pannimedu	50.0	0	0	0	0	0	0	0	0
Puthuthottam	0	28.0	48.0	44.0	56.0	0	0	8.0	16.0
Sivamalai	18.2	0	9.1	9.1	27.3	9.1	9.1	0	0
Andiparai	0	83.3	16.7	50.0	50.0	16.7	0	0	16.7
Urulikkal	33.3	0	0	11.1	33.3	0	0	0	0
Shekkalmudi	42.9	0	0	14.3	0	0	0	0	0
Akkamalai	70.0	0	0	40.0	10.0	0	0	0	0

### Number of Taxa Per Individual

The mean number of parasite taxa per individual for a group, which varied from 0.57 to 2.43, showed no relationship with fragment area (*r_s_* = −0.333, P = 0.381) but was correlated with group size (*r_s_* = 0.741, P = 0.023) and was significantly higher in fragments with human settlements nearby (Figure 1b). Even when the effect of group size was normalized for, groups close to human settlements still had a significantly higher number of parasite taxa per individual (F = 6.12, P = 0.048).

### Number of Taxa Per Group

The total number of taxa per group, which varied from 1 to 6, was not correlated with the number of samples collected (*r_s_* = 0.437, P = 0.239), fragment area (*r_s_* = 0.341, P = 0.369) or group size (*r_s_* = 0.548, P = 0.127). The groups near human settlements had significantly higher number of parasitic taxa (mean = 5.6±0.245, standard error; Figure 1c) than those away (mean = 2.5±0.5, Mann-Whitney U = 0.0, P = 0.016). Once the effect of human settlement was normalized (F = 32.372, P = 0.002), larger groups had more parasitic taxa (F = 12.267, P = 0.017).

### Overall Prevalence

The prevalence of parasites in a group varied considerably, from 42.9% to 100% (Figure 1d). Among the three covariates that we examined, prevalence strongly correlated with group size (*r_s_* = 0.742, P = 0.022), but not with fragment area (*r_s_* = −0.234, P = 0.544), the difference between groups near and away from human settlement being significant (Mann-Whitney U = 2.0, P = 0.049).

### Inter-taxa Differences

**Figure 2:**
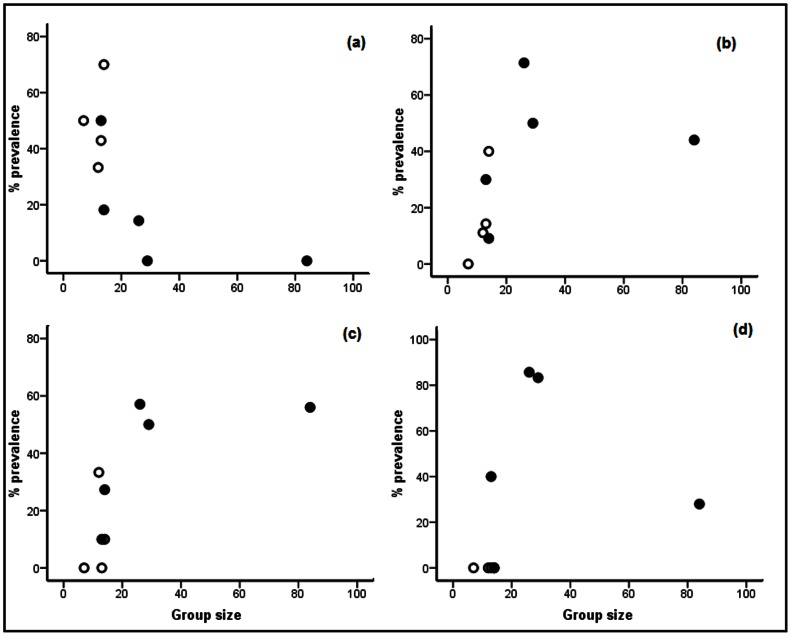
The relationship between parasite prevalence and group size in the lion-tailed macaque in nine forest fragments; (a) *Ancylostoma*, (b) *Strongyloides*, (c) *Trichuris* and (d) *Bunostomum*; fragments (•) with or (O) without human settlements.

As many as 6 out of the 9 taxa that we recorded occurred only in fragments near human settlements, although all of them did not occur in all such fragments (Table 2). *Ancylostoma* had a higher prevalence in fragments away from human settlements (49.1±7.8%; Figure 2a) than those close to them (16.5±9.2%). *Trichuris, Strongyloides* and *Bunostomum* had higher prevalence in fragments near human settlements (40.1±9.3%, 40.9±10.4% and 47.4±16.5 respectively; Figure 2b, c & d) compared to fragments away from human settlements (10.8±7.9%, 16.4±8.5%, and 0.00% respectively). Prevalence of *Ancylostoma* decreased as group size increased (*r_s_* = 0.649, P = 0.059), while that of *Trichuris* (*r_s_* = 0.667, P = 0.050) increased and that of *Strongyloides* did not seem to show any pattern (*r_s_* = 0.461, P = 0.221). The linear models showed both proximity to human settlements and group size to significantly influence prevalence of the three taxa, as the 95% confidence intervals of their coefficients did not include zero (Table 3). As is evident from the slopes, the prevalence of *Ancylostoma* was higher in smaller groups in fragments away from human settlements, while both *Trichuris* and *Strongyloides* showed an opposite pattern.

**Table 3:** Parameter estimates from linear regression models for the influence of human settlements (presence/absence) and group size on prevalence of three groups of gastrointestinal parasites of lion-tailed macaque.

Covariates	*Ancylostoma*	*Strongyloides*	*Trichuris*
Human settlement	23.570 (22.07 to 25.07)	−19.163 (−20.664 to −17.662)	−19.580 (−21.081 to −18.080)
Group size	−0.414 (−0.447 to −0.381)	0.248 (0.215 to 0.282)	0.446 (0.412 to 0.479)

95% Wald confidence intervals are given in parenthesis.

## Discussion

This is the first report on the impact of habitat fragmentation on gastrointestinal parasites in lion-tailed macaques in fragmented rainforest habitat in Anamalai Hills, Western Ghats. We found nine parasite taxa, groups near human settlements had greater prevalence and number of taxa, and these variables also had significant positive correlation with group size. Furthermore, we also found these parameters were also greater in groups near human settlement after controlling for group size. The prevalence of parasites in the lion-tailed macaque populations was low compared to other primates elsewhere. Gotoh [26] reported that 80% of fecal samples were infected with gastrointestinal parasites in Japanese macaques from 14 natural habitats studied. In Tana river, colobus monkeys had 76.6% of prevalence, which is comparable to present study [11]. In another worldwide study on primates threatened species had a lower prevalence of parasites (15.3%) than non-threatened species (19.1%) [27]. Infection of directly transmitted parasites was positively correlated with host density, group size and social nature of the hosts [28]. Host density is considered as the most important factor affecting directly transmitted parasites and its prevalence and diversity [29], [30]. In this study, a significant correlation between group size and prevalence of parasite was observed but this relationship was further influenced by the presence of human settlements. The presence of human settlements in forest areas in India is invariably also associated with the presence of livestock [31]; this was the case in the Anamalai Hills also, with all fragments with human settlements reporting the presence of livestock. Many macro-parasites can have multiple host species of different taxa [32]. In fact, 6 out of 9 taxa that we found in the lion-tailed macaque (*Bunostomum, Haemonchus, Strongyloides, Cotylophoron, Moniezia* and *Balantidium*) have ungulates as their definitive host, while 2 (*Ancylostoma* and *Trichuris*) have felids, canids and humans as definitive hosts and *Diphyllobothrium* has most mammals as its definitive hosts [23], [25]. Therefore, much of the impact of human settlements on parasitic profile of the lion-tailed macaque is most likely through the livestock which regularly graze in the adjoining forest fragment.

*Strongyloides* infection is common among human and non-human primates and other animals including cattle. It has both direct and indirect life cycles, and the third stage larvae penetrate the skin or oral mucosa to enter the host [33], [34]. High intensity of infections might cause diarrhea and weight loss. In humans, long-term infection leads to fibrosis of the intestine [35]. In baboons, a very high prevalence of infection was observed in younger animals compared to adults [36]. In the present study, we found this species in most of the forest fragments and its prevalence was significantly higher near human settlement indicating a high rate of transmission between human/cattle and lion-tailed macaque. With severe infection these macaques might be facing the same pathological problems as humans.

*Trichuris* infection is also a common parasite in human and in other mammals. It is non pathogenic and transmitted through direct ingestion of first-stage infective larva. Its eggs hatch when a suitable host ingests them [33]. This is another highly prevalent parasite in the present study, found in fragments where human activities were also observed indicating a high level transmission from human and other animals including cattle.

*Ancylostoma* (hook worm) has a direct life cycle with no intermediate host. Its infective eggs enter through water or by direct penetration through the skin. Infection causes anemia, and severe chronic infection leads to retardation in growth and development [37]. Except two forest fragments, all the groups had higher prevalence of this parasite, indicating that these populations are under severe stress from hook worms. This parasite had higher prevalence in groups away from human settlements and in smaller groups, a pattern different from all other parasites.

*Balantidium* was found only in two forest fragments in the present study. It is pathogenic, causes diarrhea and dysentery [38] and is transmitted through contaminated water.

*Bunostomum* is commonly found in ungulates and other small ruminants. It has a direct life cycle and is transmitted through the skin or contaminated water. Severe infection causes diarrhea and weight loss [38]. In the present study, four groups were infected and these fragments had a human settlement nearby.

*Cotylophoron* is common in cattle, sheep and goats of India. It has an indirect life cycle and is transmitted through contaminated water. Severe infection leads to hemorrhage and anemia [39]. This trematode was found in four fragments where cattle and goat grazing was common.

*Haemonchus* is a nematode parasite commonly found in sheep, goat, cattle and wild ruminants. It is transmitted through ingestion of third stage infective larva. This worm has an indirect cycle and its immature forms develop in mites. Severe infection leads to anemia and death [40]. This nematode was found in two forest fragments where cattle and human settlements were present.

Parasites such as *Strongyloides*, *Trichuris* and *Ancylostoma* occurred in more fragments and their prevalence was higher near human settlement and was further influenced by the group size, *Ancylostoma* declined, while the others increased with increase of group size. Of the nine taxa, six were found only in fragments near human settlements and they were primarily ungulate/human parasites. Most of these parasites have a direct life cycle which would facilitate their transmission easily. All the rare taxa had low prevalence and were found only near human settlements. This is an indication of invasion following disturbance to the natural habitat. Degraded and disturbed habitats are more likely to harbor more species and have higher prevalence than undisturbed habitats [41], [42]. Fragments such as Puthuthottam, Andiparai, Korangumudi and Varattuparai were under serious anthropogenic pressures including cattle grazing throughout the year.

Increasing host density increases the probability that a given infective egg/cyst will contact a host and thus host density should increase the parasite species richness [43]. In the present study, we found host density to be a major determinant of parasite prevalence and species richness in the lion-tailed macaque. Due to lack of dispersal, group sizes in small and isolated fragments are often higher, which increases the host density in a restricted environment [16]. The present finding supports the hypothesis that high host density mediates increased parasite prevalence and richness due to habitat fragmentation among the primates [44], [10].

Intensity of parasite infection and prevalence are also correlated with environment contamination including soil because the parasites need to develop in the environment before becoming infective. Six out of 9 fragments had human settlement, public road or both. This provided easy access to people and livestock- major sources of invading parasites. The occurrence of several rare parasitic taxa in the lion-tailed macaque in such fragments, but not in the others, is a clear indication of invasive species. Common water sources often increases exposure of infective stages of various parasites of human and domestic animal feces [42], [45] and intensity of infection can change with accessibility of water resources [46]. This may be true in case of Puthuthottam, Korangumudi, Varattuparai and Andiparai where stream water was shared extensively by human, cattle and monkeys, thereby increasing direct contact which intermediate hosts.

The health and nutrition of the host play a major role in parasitic infection and diseases [47] by directly influencing immune responses and acquisition of immunity against the parasites [48]. Healthy and well fed animals are able to cope up with parasite load and infection [49]. While the scarcity of food directly increases the stress in primates and the stress has been shown to increase the biological significance of parasite infections in colobus monkey [28]. Similarly, we have earlier reported that monkeys in degraded fragments such as Puthuthottam, Varattuparai and Korangumudi eat less nutritious food compared to those in contiguous forests [50]. Therefore lion-tailed macaques in these fragments might have been under nutritional stress thus making them susceptible to invading parasites. Overall the present study suggests that high prevalence and species richness are directly related to habitat fragmentation, high anthropogenic activities and high host density. The present findings would partially explain the reasons for the declining of immature survival in lion-tailed macaque populations in small and degraded forest fragments in the study area [16].

## Materials and Methods

### Study Sites

**Figure 3:**
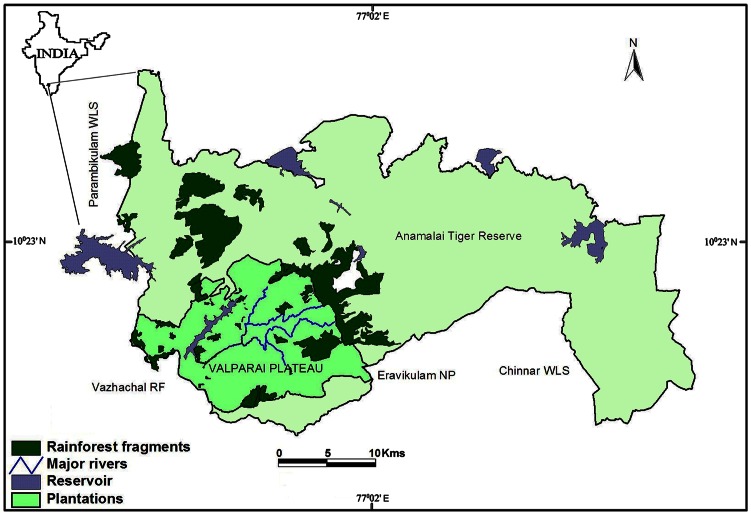
Rainforest fragments in Anamalai Tiger reserve, Western Ghats, India.

This study was conducted in rainforest fragments in the Valparai plateau, in the Anamalai Hills [16], [19]. Rainforest, which once covered the 220 sq. km plateau, was mostly clear-felled for tea, coffee and cardamom plantations between 1890s and 1930s leaving behind several forest patches ranging from 2 ha to 2000 ha in area [20] (Figure 3). The patches occur in a matrix of tea and coffee plantations at an average elevation of 1000 m ASL, and many of them have settlements of estate workers at the edge. The annual rainfall in the area is about 3000 mm, with 80% of the rainfall during the southwest monsoon in June-September, with a peak in July. The northeast monsoon (October-November) accounts for most of the remaining rainfall. The original vegetation in the area was tropical rainforest, dominated by *Cullenia-Palaquium-Mesua* association [21]. However, many of the fragments are partly under-planted with coffee and have undergone repeated selective logging for timber and shade management. Most of the small fragments are privately owned and the larger fragments belong to the State Forest Department. Due to the low incidence of poaching, this fragmented landscape has retained much of its original biota which includes large herbivores such as elephant, gaur and sambar, large carnivores such as tiger, leopard and wild dog, arboreal mammals such as the lion-tailed macaque, Nilgiri langur and Malabar giant squirrel, and several species of endemic amphibians, reptiles and birds [22]. About 20 groups of lion-tailed macaques occur in these forest patches and most of them are isolated as one or two groups in each fragment [16]. The largest fragment, about 2000 ha in area, has more than 5 groups.

We collected fecal samples from one group each in nine forest fragments, between January and March 2010. Samples were collected immediately after defecation mostly from identifiable individuals, thus avoiding repeated collections. Collection was carried out without direct interaction with the macaques and causing no disturbance to the habitat. Permission to collect lion-tailed macaque fecal samples from Anamalai Tiger Reserve and from privately owned forest fragments was granted by the Principal Chief Conservator of Forests and Chief Wildlife Warden, Tamil Nadu State Forest Department, Chennai 600 015 (Letter Ref. No. WL 5/58890/2008, dated 2nd September 2009). Private estate owners were informed in person about the collection of samples. Samples were stored in 15 mL falcon tubes in 10% formalin solution and transported to the laboratory. Samples were examined for helminth eggs and larvae and protozoan cysts after concentration using sodium nitrate floatation and sedimentation techniques [23], [24]. We prepared slides using the above methods for microscopic examination of parasites, counted and identified parasites up to genus level on the basis of egg, larvae, cyst coloration, size, shape and contents [23], [25]. We used iodine to identify the protozoan and an ocular micrometer to measure the size of eggs and cysts. From this data we estimated the following response parameters for each fragment:

1. Prevalence: the percentage of fecal samples with any parasite taxon.
2. Number of taxa: the number of different parasitic taxa recorded per individual and for all samples together from a group.

The covariates that we recorded for each fragmented included fragment area estimated from a digitized map available for the study site, the presence of livestock in the fragment, the presence of human settlement adjacent to the fragment and the number of animals in the study group. The tree density, tree basal area and canopy cover in forest fragments in the study site have been reported to be strongly and positively correlated with fragment area [16], [18]. Therefore, we did not include these as covariates of the parasitic profile of the lion-tailed macaque.

### Data Analysis

We used Mann-Whitney U statistic to test for differences between two samples and Spearman rank correlation coefficient (*r_s_*) to examine association between two variables. We used linear regression model to identify the covariates that significantly influenced the prevalence of different parasite taxa. A covariate was considered significant only if the 95% confidence interval of its coefficient did not include zero. We used SPSS (v. 17.0) for statistical analyses.
